# *Halomonas* sp. MC140, a polyhydroxyalkanoate (PHA) producer isolated from the Arctic environment

**DOI:** 10.1038/s41598-025-06898-7

**Published:** 2025-07-03

**Authors:** Mikkel Christensen, Iulia Chiciudean, Irina Lascu, Piotr Jablonski, Volha Shapaval, Boris Zimmermann, Ana-Maria Tanase, Hilde Hansen

**Affiliations:** 1 Department of Chemistry, UiT The Arctic University of Norway, 9037 Tromsø, Norway; 2 Faculty of Science and Technology, Norwegian University of Life Sciences, 1432 Ås, Norway; 3 Department of Genetics, Faculty of Biology, University of Bucharest, 050095 Bucharest, Romania; 4 Department of Chemistry, Umeå University, 90187 Umeå, Sweden

**Keywords:** PHA, Polyhydroxyalkanoates, PHB, PHBV, *Halomonas*, Biolog, Bacteria, Bacterial genetics, Bacterial genomics, Marine microbiology, Biomaterials, Biopolymers

## Abstract

Polyhydroxyalkanoates (PHA) are biobased and biodegradable polyesters with potential to replace petroleum-based plastics or for use as feed in aquaculture. In this study, *Halomonas* sp. MC140 was isolated from the Arctic littoral environment in Norway and characterized in terms of PHA production. Phylogenetic analysis of the draft genome sequence placed the strain as closely related to *Halomonas profundi* MT13. A genome-based model of PHA biosynthesis pathways was constructed, and shake-flask experiments identified the highest PHA production in the form of polyhydroxybutyrate (PHB) with acetate (35 ± 4.8%) or glucose (28 ± 7.9%) supplementation, as verified by gas chromatography-flame ionization detector (GC-FID) analysis, while the addition of propionate produced PHB-co-valerate (PHBV) co-polymer (≈ 1% 3-HV). Low PHB production (< 5%) was found from supplementation with glycerol or fructose. A multivariate Partial Least Squares Regression (PLSR) model for low- to medium-range (0–40%) PHB content in *Halomonas* sp. MC140 was constructed with Fourier transform infrared spectroscopy (FTIR) spectra and used to assess PHA production over time. Additionally, the strain’s carbon substrate utilization phenotype was characterized using the Biolog assay. These findings suggest that PHA production by *Halomonas* sp. MC140 is modest compared to other *Halomonas* species but provides a starting point for optimization or use in sustainable feed applications in Norwegian aquaculture, where lower PHA production is sufficient compared to bioplastic production.

**Supplementary Information:**

The online version contains supplementary material available at 10.1038/s41598-025-06898-7.

## Introduction

Bacterial production of PHA is attracting attention as part of a sustainable bio-based industry. The PHA polymers can be used as biodegradable bioplastics or in their monomeric form as platform chemicals. Polyhydroxyalkanoates are polymers composed of hydroxyalkanoic acid (HA) monomers connected by ester-bonds. The HA monomers contain a stereogenic center in (*R*)-configuration with a side group typically being an alkyl group located at the 3-carbon position. The length and composition of the side group varies and determines the type of PHA and causes differences in material properties for the different types. The number of carbon atoms in the hydroxylated monomer forms the basis for classification of PHA as short-chain length (scl)-PHA (C_3_–C_5_), medium chain length (mcl)-PHA (C_6_–C_14_), or long chain length (lcl)-PHA (C_15_ or higher)^1,2^. The chain length and composition of co-polymers are determined by organism specific pathways (e.g., glycolysis, tricarboxylic acid- and the propionate cycle) and the presence of specific PHA biosynthesis- and synthase genes in various bacteria^3^. The PHA biosynthesis pathway can be activated by the expression of only three enzymes: the ß-ketothiolase acetoacetyl-CoA acetyltransferase PhaA, the acetoacetyl-CoA reductase PhaB, and the PHA synthase PhaC. This pathway starts from the universal and ubiquitous precursor acetyl-CoA and continues in a sequential condensation reaction of two acetyl-CoA molecules by PhaA, followed by reduction to (*R*)-3-hydroxybutyryl-CoA by PhaB and polymerization into PHB by PhaC^4^. A second type of ß-ketothiolase, *Re*bktB, is found in *Cupriavidus necator* (syn. *Ralstonia eutropha*), the model organism for PHA production. While PhaA primarily utilizes acetyl-CoA (C_2_) as substrate, *Re*BktB also has substrate affinity for propionyl-CoA (C_3_) and butyryl-CoA (C_4_)^5^. Thus, *Re*BktB can produce PHAs of chain length C_4_, C_5_ and C_6_ through one or more condensation reactions, while PhaA produces the C_4_ precursor acetoacetyl-CoA, the substrate for PhaB.

The Gram negative Gammaproteobacteria *Halomonadaceae* family was described by Frantzmann in 1988^6^. The family includes the *Halomonas* genus, which was described by Vreeland and co-workers after isolation of the extremely salt-tolerant type strain *Halomonas elongata* 1H9 from condensers located at a solar salt facility^7^. The *Halomonadacea* comprise the largest family of halophilic bacteria and has undergone several reclassifications through the years. Most recently, a comprehensive phylogenetic analysis was conducted by de la Haba using whole genome sequencing (WGS) data^8^. This phylogenetic revision suggests that *Halomonas*, which is the only non-monophyletic group identified in the WGS analysis, should be split into seven genera: *Halomonas sensu stricto*,* Vreelandella*,* Bilgrantia*,* Franzmannia*,* Litchfieldella*,* Onishia*, and *Modicisalibacter*. These seven genera comprise more than 100 species with a valid name, of which 38 species are proposed classified as *Halomonas sensu stricto*^9,10^. Many of these strains are halophilic, but most are halotolerant and grow in media without- or with low salt concentration^11,12^. Marine *Halomonas* strains are particularly relevant for industrial PHA production due to natural production of PHB^13^, PHBV^14^, and co-production of PHA with other biomolecules such as ectoine^15^. *Halomonas* strains isolated from marine Arctic and sea-ice environments also found PHA producers, including a strain that may naturally produce mcl-PHA^16^. Precise genetic engineering tools such as CRISPR/Cas9 have furthermore been used with success in *Halomonas bluephagenesis* TD01 to increase the fraction of 3-hydroxyvalerate (3-HV) in PHBV^17^, to hyperproduce the 4-hydroxybutyrate constituent of P3HB-co-4HB^18^, and to tailor the PHA composition to contain mcl-PHA^19^ or the non-natural 5-hydroxyvalerate^20^.

Production of PHA from renewable (i.e. sustainable) resources is desirable and may reduce environmental and economic cost of industrial PHA production^21^. For this purpose, waste streams such as glycerol from biodiesel production and biological substrates such as hydrolyzed algae have been used as sustainable carbon sources for PHA production in *Halomonas*^22,23^. Bacteria containing PHA also showed potential in sustainable food production, serving as feedstock and as an alternative to antibiotics in aquaculture^24^. This potential has also been demonstrated for a PHB-producing *Halomonas* isolate used as feed for White shrimp, an economically important species farmed in China^25^.

The use of extremophilic bacteria, such as halophilic *Halomonas*, for bio-industrial production has been termed “*Next generation industrial biotechnology*”^26^. Characterization of *Halomonas* strains that produce PHA by means of genotyping, chemical analysis, and substrate utilization, serves as a first step for developing the bio-industrial potential of members of this fascinating genus.

The purpose of this study was to determine the PHA-producing potential of a new strain,

*Halomonas* sp. MC140, isolated from the Arctic region of Norway. To achieve this, we used a genome sequence-guided approach combined with phenotypic assays to characterize PHA production from supplementation with various carbon substrates. These analyses were complemented by chemical characterization using multivariate FTIR and GC-FID analyses, including Principal Component Analysis (PCA), which enabled the construction of a prediction model using Partial Least Squares Regression (PLSR) for low- to medium-range (0–40%) PHA content in *Halomonas* sp. MC140 based on FTIR spectra.

## Results and discussion

The isolate *Halomonas* sp. MC140 was obtained from an environmental swab collected from a maturation room for salted fish at a fish-landing facility near Tromsø, Norway, as part of a previously described study to find PHA producing bacteria from the Arctic littoral environment^27^. Bacterial colonies were circular with regular borders, convex, and opaque creamy white when observed on Marine agar (MA) plates.

The isolate appeared as a potential producer of PHA as determined by presence of a low intensity broad carbonyl-ester band in the 1720–1750 cm^− 1^ region in a FTIR spectrum obtained from colonies grown on MMY agar plates supplemented with glucose^28^. In contrast, colonies sampled from MA plates did not show any peaks in this region (Supplementary Figure S1).

The ability to produce PHA is a common feature of *Halomonas*, and WGS was performed to identify potential PHA biosynthesis genes and to resolve the phylogenetic placement in depth. The PHA production potential was subsequently characterized by supplementing four carbon substrates independently and testing co-addition of propionate to 50 mL shake-flask cultures.

### Strain identification by 16S rRNA gene and WGS reveals closest match to *H. profundi* MT13 (*Vreelandella* cf. *profundi*)

The genome of *Halomonas* sp. MC140 was sequenced using Illumina paired-end sequencing of 301 bases in both directions. The annotated genome contained 3497 genes, of which 3391 were predicted to be protein-coding sequences. The main characteristics of the annotated genome are presented in Supplementary Table S1. Mapping of reads against the assembly showed that 83.7% and 99.4% of the raw and trimmed reads, respectively, were mapped onto the genome. The genome assembly was assessed to be of acceptable quality by evaluation against Busco’s *Oceanospirillales* database, which found only three genes were missing, one was fragmented, and 614 genes were complete (out of 619 genes).

**Fig. 1:**
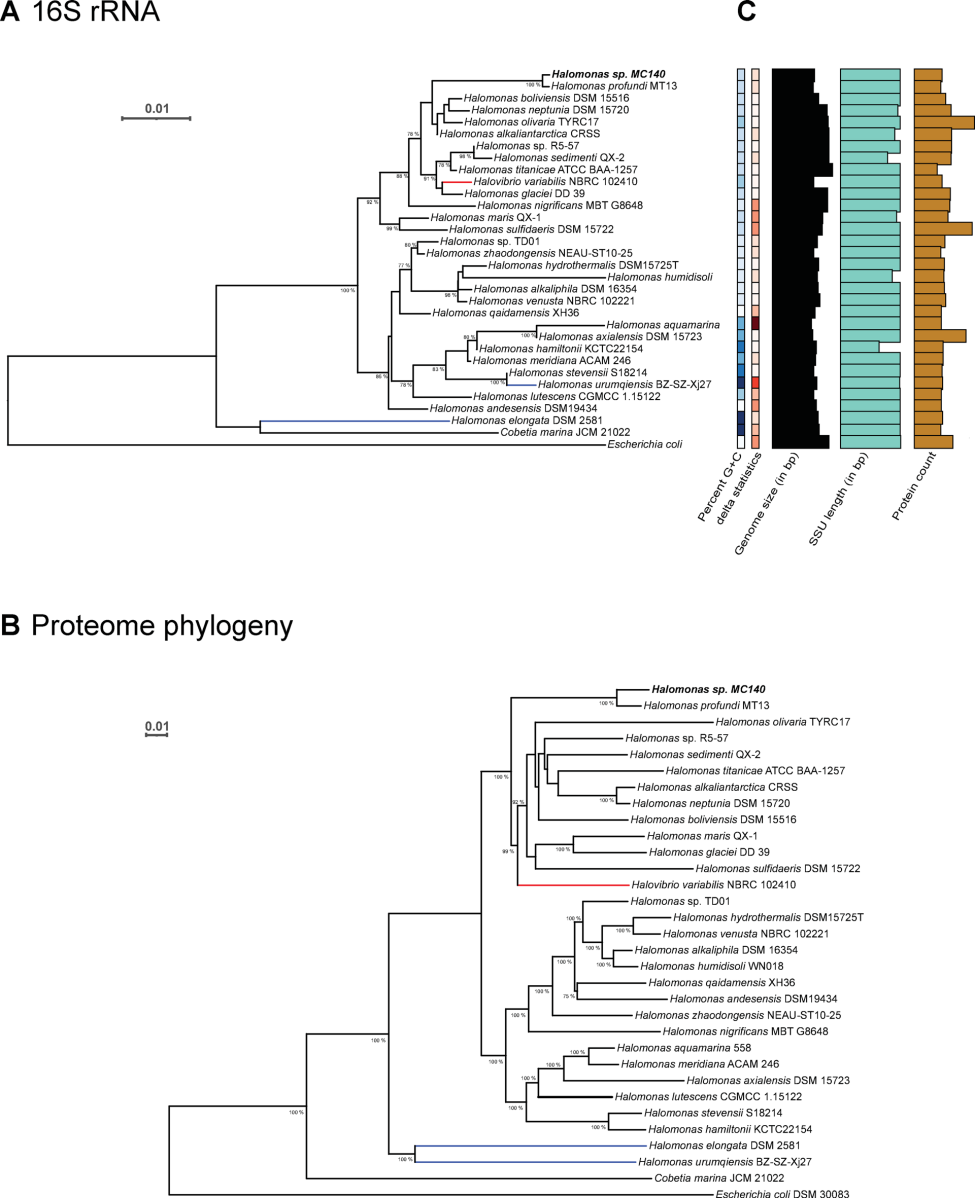
*Halomonas* sp. MC140 is most closely related to *Halomonas profundi* MT13. (**A**) Phylogeny inferred from GBDP distances calculated from 16S rDNA gene sequences. The numbers above branches are GBDP pseudo-bootstrap support values > 70% from 100 replications, with an average branch support of 73.4%. The black *Halomonas* branches belong to the newly suggested genus *Vreelandella*, while the red branch indicates the suggested non-Halomonadacea *Halovibrio variabilis* and the blue branches are part of *Halomonas sensu stricto*^8^. *Cobetia marina* and *Escherichia coli* serve as outgroups. (**B**) Phylogeny inferred from the translated whole-proteome-based GBDP distances. Branch values are GBDP pseudo-bootstrap support values > 70% from 100 replications, with an average branch support of 90.7%. (**C**) The “percent G + C” and “delta statistics” are represented by colors grouped by similarity as clustered by TYGS. The length of the “genome size”, “protein count” and “16S rRNA small subunit (SSU)” bars are depicted by their length for comparison between species.

The complete 16 S rRNA sequence obtained from the annotated genome was used alongside the translated proteome to construct two phylogenetic trees via DSMZ’s Type (Strain) Genome Server (TYGS), as detailed in the Methods section. The resulting phylogenetic trees, shown in Fig. 1A,B, include the genus type-strain *H. elongata* and the Arctic isolate *Halomonas* sp. R5-57, selected based on prior studies of PHA production^29,30^, and *Cobetia marina* and *Escherichia coli* as outgroups. The remaining species included in the phylogenetic trees were selected by the TYGS pipeline.

Both of the phylogenetic trees (Fig. 1A,B) show that *Halomonas* sp. MC140 group most close to *H. profundi* MT13 (*Vreelandella* cf. *profundi*). The Arctic isolate *Halomonas* sp. R5-57 is positioned two nodes higher in the 16 S rRNA phylogenetic tree and one node higher in the proteome-based tree relative to *Halomonas* sp. MC140. It can also be observed that *Halomonas* sp. MC140 has a slightly smaller proteome compared to *Halomonas* sp. R5-57, as illustrated in Fig. 1C. According to the TYGS analysis, *Halomonas* sp. MC140 might qualify as a new species, but a more thorough biochemical study is needed to establish if it separates from *H. profundi* MT13 on the species level^12,31^.

The phylogeny was furthermore compared with the recently published phylogeny by de la Haba, which suggests the forming of several new genera from splitting of *Halomonas*^8^. It was found that all *Halomonas* strains included in our study belong to the newly suggested genus *Vreelandella*, except for *H. elongata* and *Halomonas urumqiensis* which belong to *Halomonas sensu stricto*^8^. In our 16 S rRNA tree, *H. urumqiensis* is placed in the *Vreelandell*a clade, while *H. elongata* is placed on a separate branch. When looking at the proteome tree, *H. elongata* and *H. urumqiensis* are placed on the same branch and thus separate from the *Vreelandella* clade. The inclusion of *Halovibrio variabilis*, however, makes the phylogeny polyphyletic, as it groups within *Halomonas* in both trees. *Halovibrio* has previously been subject to discussions about its potential classification as *Halomonas*^32^ and recent evidence suggests it to be placed outside the *Halomonadacea* family, although further studies are warranted^8^.

The guanine (G) and cytosine (C) content of bacterial DNA is a highly variable metric between different bacterial species^33^. The GC content of bacterial DNA is therefore commonly reported as part of strain descriptions and used for phylogenetic analyses. The GC content of 54.1% of *Halomonas* sp. MC140 is close to that of *H. profundi* MT13 and in the lowest range of the *Halomonas* species analyzed, as indicated by the color markings shown in Fig. 1C. It is furthermore clear from the GC content bar that *Halomonas sensu strictu* and *C. marina* have the highest GC contents with *H. elongata* DSM2581 showing 63.6% GC, which is the highest of the analyzed species (Fig. 1C).

The accuracy of the 16 S tree was assessed by the TYGS pipeline and evaluated with delta statistics (Fig. 1C), where a lower delta value indicates a stronger tree-like structure and greater branch support^34^. *Halomonas variabilis* NBRC 102,410 and *Halomonas hydrothermalis* DSM15725^T^ had the lowest delta values (0.305), represented by white color in Fig. 1C, whereas *Halomonas aquamarina* 558 had the highest (0.496), shown in dark red. Genome sizes varied from 3.5 Mb in *H.aquamarina* 558 to 5.3 Mb *Halomonas titanicae* ATCC BAA-1257, depicted by black bars in the figure. The number of calculated protein sequences also differed significantly, ranging from 2874 in *H. titanicae* ATCC BAA-1257 to 7375 in *H. olivaria* TYRC17. Surprisingly, the relatively large genome of *H. titanicae* ATCC BAA-1257 confers the lowest number of calculated protein coding sequences.

Based on these analyses, our phylogeny appears to be in concordance with the recently published comprehensive *Halomonas* phylogeny and identifies *Halomonas* sp. MC140 as most closely related to *H. profundi* MT13. This resemblance leads to interesting questions regarding the lifestyle of our strain, since *H. profundi* MT13 was isolated from deep-sea sediment (8800 m) of the Mariana Trench and shown by Electron Microscopy to produce PHA inclusions^35^. Of interest among the other closely related *Halomonas* strains is the PHA producer *Halomonas boliviensis*^36^. The close phylogenetic relationship to these PHA producers made *Halomonas* sp. MC140 a promising candidate for further studies of PHA production.

### Genome sequence-based model for PHA biosynthesis

**Fig. 2:**
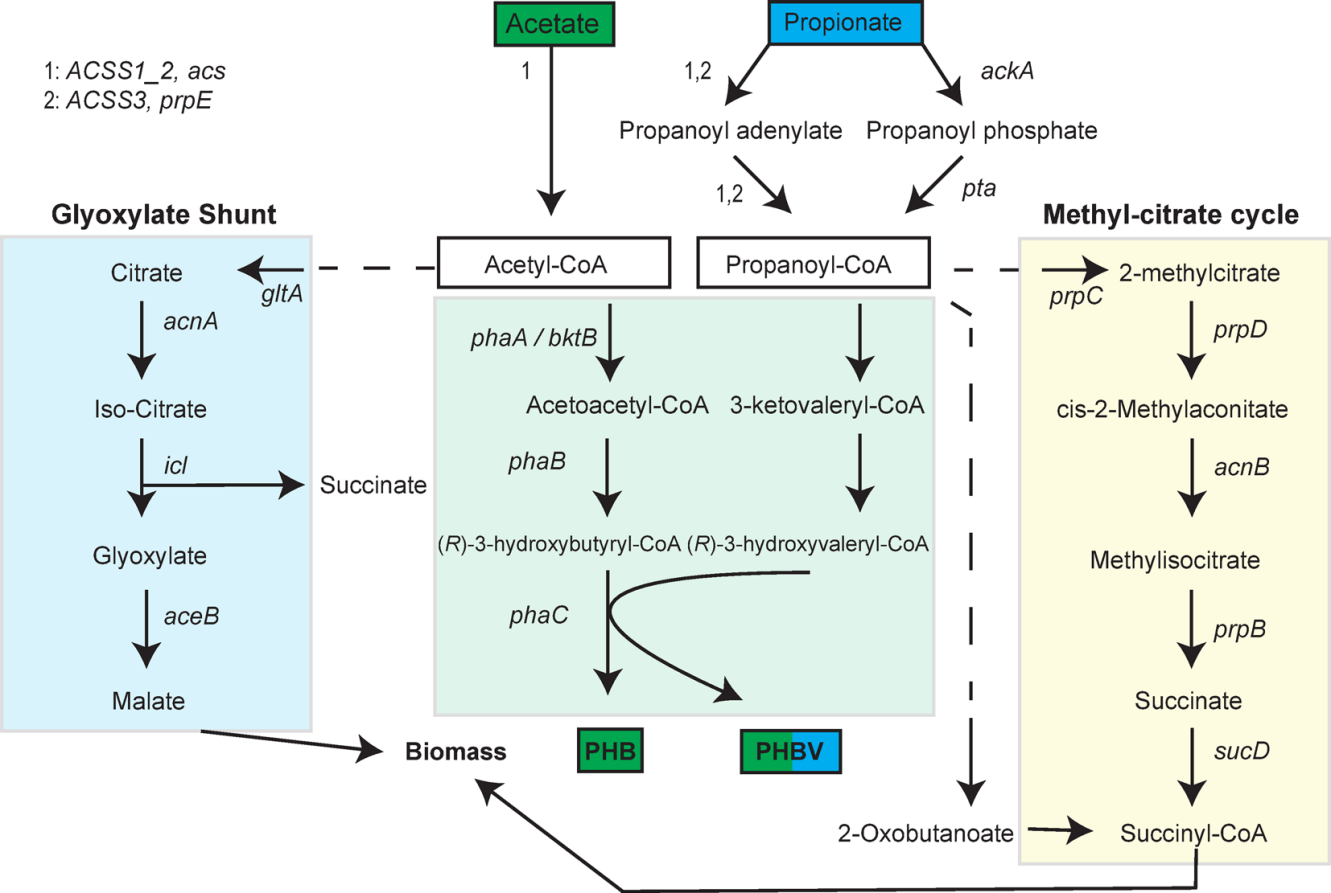
Genome sequence-based model of PHA biosynthesis in *Halomonas* sp. MC140. The biochemical pathways for production of PHB and PHBV exemplified by supplement of acetate (C_2_) with or without co-supplement of propionate (C_3_). The glyoxylate shunt and the methyl-citrate cycle are alternative pathways that may direct PHA precursors towards other forms of biomass production.

The PHA biosynthesis genes (*phaA*/*bktB*, *phaB*, and *phaC*) required for PHA production were identified in the annotated genome, as well as PHA regulatory- and degradation genes (Table 1). To construct a genome sequence-based model for PHB and PHBV production (Fig. 2), the genes involved in propionate metabolism, methyl citrate cycle and the glyoxylate shunt were also identified (Supplementary Table S2). As illustrated in Fig. 2, a carbon substrate such as acetate is converted to acetyl-CoA, the universal C_2_ precursor for scl-PHA. Co-supplement with propionate can additionally provide a C_3_ precursor which is further converted to propanoyl-CoA via the *ackA*/*pta* or the *ACSS1/2/3* route. Interestingly, two acetyl-CoA acyl- and acetyltransferases (*PhaA)* and a beta-ketothiolase (*bktB*) were annotated. This suggests that *Halomonas* sp. MC140 may be able to incorporate propionyl-CoA into valeryl-CoA, based on what is known about *Re*bktB from *R. eutropha*^37^.

**Table 1:** PHA biosynthesis genes annotated in the genome of *Halomonas* sp. MC140.

Pathway	Gene(s)	Protein function (EC.)	KEGG id	Locus tag	AA identity (%)
PHA biosynthesis	*bktB*	Beta-ketothiolase	K00626	JNO04_16210	97.5
	*phaA*	Acetyl-CoA C-acyltransferase (1: 2.3.1.16, 2: 2.3.1.9)	K00626	1: JNO04_11225 2: JNO04_13785	1: 99.0 2: 100
	*phaB*	Acetoacetyl-CoA reductase (1.1.1.36)	K00023	JNO04_00095	99.6
	1: *phaC*_*1*_ 2: *phaC*_*2*_ 3: *phaC*	Polyhydroxyalkanoate synthase enzyme (2.3.1.304)	K03821	1: JNO04_10760 2: JNO04_10170 3: JNO04_15010	1: 97.9 2: 97.5^a^ 3: 96.3
PHA regulation	*phaR*	Repressor		JNO04_10625	98.6^b^
	*phaP*	Phasin family protein		1: JNO04_10755 2: JNO04_12385 3: JNO04_15380	1) 99.3 2) 99.2 3) 97.6
PHA degradation	*phaZ*	PHA depolymerases (3.1.1.75 and 3.1.1.22)		1: JNO04_05475 2: JNO04_07145 3: JNO04_05475	1: 95.7 2: 92.0^c^ 3: 95.7
	*scoA/B*	3-oxoacid transferases A/B (1/2: 2.8.3.5)	1: K01028 2: K01029	A: JNO04_08835 2: JNO04_08830	1: 99.6 2: 100^d^
	*bdh*	1: 3-hydroxybutyrate dehydrogenase (1.1.1.30) 2: Hypothetical protein	K00019	1: JNO04_08815 2: JNO04_01265	1: 98.8 2: 71.7^e^

The amino acid (AA) identity is relative to *H. profundi* MT13, except a: *Halomonas neptunia* b: *Halomonas* sp. JB37 c: Halomonas spp d: H. profundi e: *Halomonas* sp. HAL1.

The second gene required in the scl-PHA biosynthesis pathway *PhaB* encodes an acetoacetyl-CoA reductase. To our knowledge, the only biochemically characterized PhaB from *Halomonas* is the *H. bluephagenesis* TD01 tdPhaB, which differs from PhaB found in other types of bacteria by having a cofactor-substrate specificity for reduced nicotinamide adenine dinucleotide (NADH) instead of reduced nicotinamide adenine dinucleotide phosphate (NADPH)^38^. The physiological implication of NADH preference for PhaB has been linked to increased PHB production under conditions of oxygen limitation. Alignment of the PhaB amino acid sequence from *Halomonas* sp. MC140 with tdPhaB reveals a high degree of similarity (92%) and only two conservative replacements in the substrate (NADH) binding domain: a histidine (H) to arginine (R) at position 37 and a glutamate (E) to aspartate (D) at position 40. This suggests that PHB production in *Halomonas* sp. MC140 may be higher under oxygen limitation similar to what has been observed for *H. bluephagenesis* TD01.

Three PhaC polymerase genes (*phaC*, *phaC*_*1*_ and *phaC*_*2*_*)* were annotated in *Halomonas* sp. MC140 and classified based on their lipase box domains obtained from their translated amino acid sequence (PhaC_1_: GYCLG and PhaC_2_: SYCVG) and their size (≈ 70 kDa)^27,39,40^. The putative PhaC variant differs from PhaC_1_ and PhaC_2_ by having a GNCQAG box domain and by a larger calculated weight (≈ 82 kDa). It has been reported that heterologous expression of *phaC* from *Halomonas* sp. O-1 and *Halomonas* sp. DSM2581 in *E. coli* do not lead to production of PHB^39^, indicating that the *phaC* annotation may be incorrect. However, a recent transcriptomic analysis of *Halomonas* sp. 363 showed expression of *phaC* under PHA production conditions, indicating a role in PHA production- or regulation^16^. Whether or not the *phaC* gene encodes a PHA synthase therefore remains unresolved. The presence of three *phaC* genes have also been reported in Gammaproteobacteria such as *Pseudomonas* isolated from Antarctica^41,42^.

### Production of PHB and PHBV in *Halomonas* sp. MC140

Based on the model for PHA biosynthesis (Fig. 2), production of PHA by *Halomonas* sp. MC140 seemed highly likely, potentially with PHBV or mcl-PHA production based on the annotation of *bktB*. Basic growth conditions, including temperature and salinity, were therefore investigated in order to qualitatively and quantitatively assess the PHA production potential. The highest growth rate (0.22 h^− 1^) and carrying capacity (≈ 8) were observed in LB_3 − 5_ medium at 25 °C at 200 rpm in 50 ml shake flasks, based on OD_600nm_ measurements (Supplementary Figure S2).

Production of PHA was subsequently characterized in a two-stage process, initially producing biomass in LB_3 − 5_ medium and then inducing nitrogen stress by transferring to a low-nitrogen (3.7 mM) PHA production medium. The PHA produced by *Halomonas* sp. MC140 was measured by GC-FID after methanolysis of washed and freeze-dried cells. The highest absolute PHA production was found when acetate was supplemented reaching 35 ± 4.8% PHB and a PHA productivity of 1.0 g/L PHB (Table 2). Supplementation with glucose resulted in production of 21 ± 5.5% PHB after 120 h incubation, which increased slightly to 28 ± 7.9% PHB after prolonged incubation (168 h, Table 2). More biomass was produced from glucose in comparison with acetate which resulted in a higher PHA productivity of 1.1 g/L after 168 h of incubation. Glycerol and fructose were good substrates for growth as measured by OD_600nm_ and cell dry weight (CDW), but PHA production was limited to less than 5% PHB. Based on the genome model, production of the industrially relevant PHBV co-polymer was furthermore investigated by co-supplementing propionate to glucose and acetate. Propionate appeared to have a slightly toxic (growth-reducing) effect when supplemented to both glucose and acetate, but still resulted in incorporation of approximately 1% 3-HV into PHBV (Table 2). Co-supplement with carboxylic acids of chain length C_6_ and C_8_ did not result in any mcl-PHA production.

**Table 2:** Growth and PHA production of *Halomonas* sp. MC140 from various carbon sources.production of PHA after 120 h of incubation, except after 168 h where indicated (*).

Carbon source	PHB (%)	PHV (%)	PHA (%)	OD_600nm_ (a.u)	CDW (g/L)	PHA (g/L)
Acetate	35 ± 4.8	–	35 ± 4.8	7.3 ± 1.0	2.9 ± 0.5	1.0
Glucose *	28 ± 7.9	–	28 ± 7.9	7.8 ± 2.6	4.0 ± 1.9	1.1
Glucose	21 ± 5.5	–	21 ± 5.5	6.3 ± 0.2	3.6 ± 0.8	0.8
Glycerol	4.6 ± 2.2	–	4.6 ± 2.2	4.4 ± 0.4	3.7 ± 1.4	0.2
Fructose	4.8 ± 4.0	–	4.8 ± 4.0	4.4 ± 0.1	4.2 ± 1.5	0.2
Acetate + p	25 ± 5.7	0.9 ± 0.6	26 ± 5.3	5.2 ± 1.6	2.3 ± 0.5	0.6
Glucose + p *	11 ± 3.6	1.3 ± 0.4	12 ± 3.9	5.0 ± 1.4	4.3 ± 1.2	0.5

The PHA content is reported as weight percentages (%) determined by GC-FID. Standard deviation (±) is calculated from six biological replicates obtained from two independent experiments. Optical density (OD600nm) at the time of sampling (a.u: arbitrary numbers). Propionate was supplemented where indicated (+ p).

The highest production titers of PHA by *Halomonas* sp. MC140 were relatively low compared to other *Halomonas*. In comparison, *Halomonas boliviensis* is capable of producing 54–55% PHA when grown in shake-flask cultures supplied with acetate and butyrate or glucose^36^, and the amount can increase to more than 80% when using a fermenter^36,43^. Likewise, the industrial PHA producer *H. bluephagenesis* TD01 was initially found to produce up to 69% PHB in shake-flasks with glucose as substrate, which increased to more than 80% in a 6 L fermentor^44^. High cell density fermentation furthermore shows production of more than 90% PHA in *H. bluephagenesis* TD01 from acetate^38^. *Halomonas* strains that appear to be better at converting glycerol to PHA have also been reported such as the Arctic isolate *Halomonas* sp. R5-57 reaching 17% PHB^30^ and the desert isolate *Halomonas desertis* G11 reaching 68% PHA with a small fraction of 3-HV^45^.

Production of PHA co-polymers, such as PHBV, is most commonly achieved by co-supplementing a primary growth promoting media with a secondary substrate such as propionate (C_3_). Only a few *Halomonas* strains are described to produce PHBV from a primary carbon substrate without co-supplement of propionate or valerate^16,45,46^. Production of PHBV in *Halomonas* sp. MC140 was confirmed only when propionate was co-supplemented. The 3-HV fraction (≈ 1%, Table 2) of PHBV obtained in this study is also low compared to other *Halomonas*. This may be explained by flux of propanoyl-CoA *via* the methyl citrate pathway^47^ (Fig. 2) or by use of a non-optimal concentration of propionate. By supplying propionate or valerate to glycerol, molar fractions of 12% or 39% 3-HV were achieved in *Halomonas profundus*^14^, and up to 30% 3-HV were found in *H. bluephagenesis* when the same co-substrates were supplied to glucose^44^.

### Multivariate analysis by FTIR and predictive modeling of PHA content

Fourier transform infrared spectroscopy is a non-destructive method used to chemically characterize biological systems, such as bacteria, for the presence of various functional groups^48^. Among the important bands of functional groups related to PHA is the carbonyl-ester band (C=O stretching) typically located at 1724 cm^–1^ ^27^ or 1728 cm^− 1^ for PHB^49^, or between 1732 and 1745 cm^− 1^ for mcl-PHA^49^. The peak position of this band varies with the degree of crystallinity of the PHA polymer with higher degrees of crystallinity resulting in lower wavenumber values in the FTIR spectra^50^.

The phenotypic response of *Halomonas* sp. MC140 when supplemented with the pure carbon sources (Table 2) was additionally analyzed by FTIR to complement the verification of PHA production by GC-FID analysis and to investigate the suitability of using FTIR for detection of PHA. To do so, washed samples were analyzed by FTIR at the end of the cultivation before the freeze-drying step necessary for GC-FID analysis. The resulting dataset consisted of 34 spectra, including six spectra obtained from the pre-cultures for use as zero reference, which were analyzed by PCA and used for constructing a prediction model for PHA content by use of PLS).

**Fig. 3:**
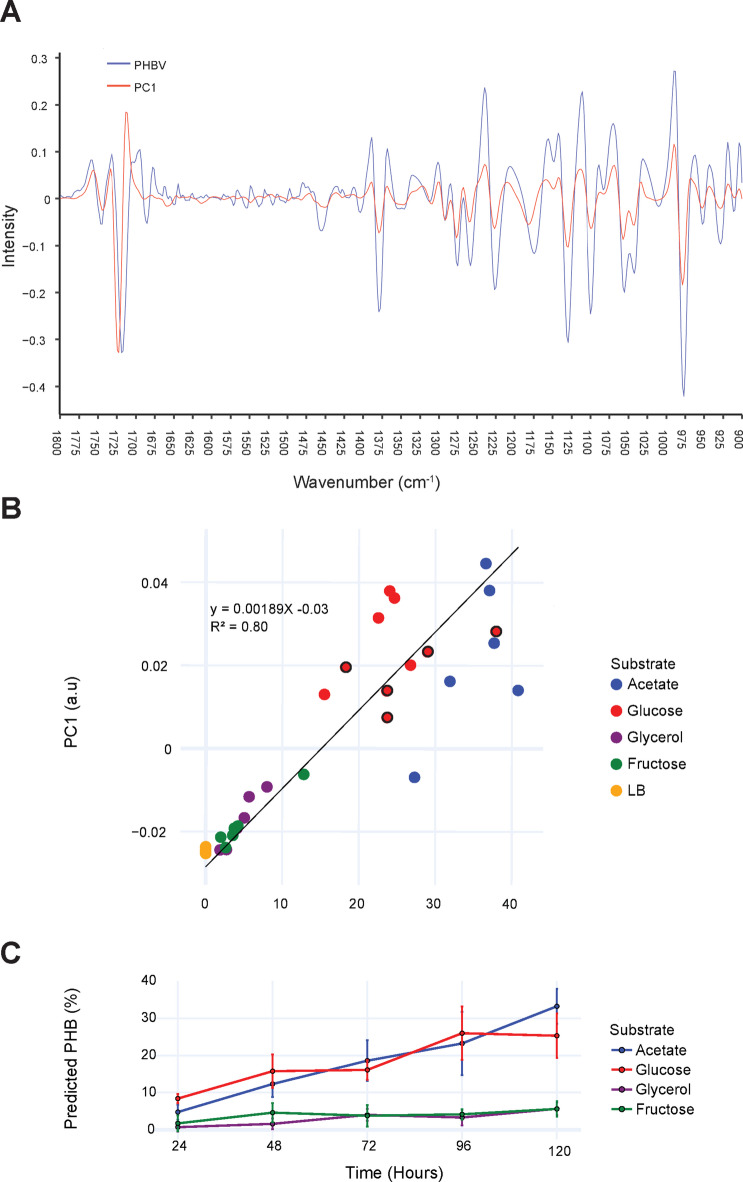
PCA analysis of FTIR spectra. (**A**) The first principal component (PC1) second derivative spectrum obtained after PCA of raw FTIR spectra (*n* = 34) overlaid with the second derivative PHBV reference spectrum. (**B**) The PCA score values of PC1 plotted against the  % of PHB in the samples supplemented with substrates indicated by color, with the linear regression relationship (R^2^ = 0.80). Spectra were sampled after 120 h and 168 h (black border on markers). (**C**) Time-course production of PHB (%) predicted by the PLSR model for the samples supplemented with four different substrates.

The PCA results showed that the first principal component (PC1) explained 86% of the variance in the FTIR dataset. Due to the derivatization applied to the spectra, strong peaks (high absorbance) in raw spectra correspond to negative peaks in the second derivative spectra. As observed from Fig. 3A, the majority of signals with high negative PC1 loadings correlate with the main FTIR absorption bands of the PHBV standard. The most notable of these signals are at wavenumbers 1379, 1276, 1259, 1130, 1100, 1056, 1042, 977, 953, and 928 cm^− 1^. Small differences in peak minima of approximately  – 5 cm^− 1^ for the PHBV standard relative to PC1 are observed at 1722 cm^− 1^ and 1182 cm^− 1^. Such shifts have previously been attributed to the degree of crystallinity of the polymer^50^, which will be slightly different when comparing a solid PHBV standard with PHB in vivo. Thus, the presence of PHB in the bacteria can be easily detected by FTIR.

A linear relationship (R^2^ = 0.80) is observed when the PC1 score is plotted against the PHB content (as measured by GC-FID), across individual replicates as shown in Fig. 3B. It is also apparent from the plot that the deviation from the linear regression line is higher in the 1st quadrant (acetate and glucose samples) relative to the 3rd quadrant (glycerol and fructose); hence, variation in the FTIR spectra increases with higher PHB content. Furthermore, PC3 versus PC1 score-plot showed that acetate and glucose samples could be differentiated based on their PC3 scores (Supplementary Figure S3). Important signals in the PC3 loadings were 978, 998, 1224, 1275, 1398, 1447, 1463, 1480, 1531, 1549, 1621, 1663, 1693, and 1711 cm^− 1^. These signals are not necessarily related to PHA but can be associated with functional groups found in carbohydrates, esters, proteins and carboxylic acids. Association of these peaks to specific compounds requires inclusion of additional reference methods.

The use of PC spectra derived from PCA for prediction based on FTIR spectra of biological samples is generally inferior to PLSR modeling. This is in part because PLSR, in contrast to PCA, maximizes and incorporates covariance between different peaks into the model, enabling better prediction.

A PLSR model was therefore constructed to demonstrate the use of FTIR for predicting the PHA content in *Halomonas* sp. MC140. To do so, the FTIR spectra were additionally normalized to amide band I^51^, before splitting into prediction (*n* = 22) and independent validation (*n* = 11) datasets. The PLSR model showed good prediction metrics as observed by correlation coefficient of determination (R^2^) of 0.96 and root mean square error (RMSE) of prediction of 4.20% for cross validation. The independent test set validation results were slightly better with R^2^ of 0.97 and RMSE of 2.26%. These validation results showed good correlation between predicted and measured PHA content (Supplementary Figure S4) and in the same range as described by others^52^. The PSLR model was subsequently used to predict the time-course increase in PHB production over time during the shake-flask cultivations when supplementing the four pure substrates. As seen in Fig. 3C, PHB production increased during the cultivation period.

These results show the potential of using FTIR for constructing a prediction model of PHA in a *Halomonas* strain with low to medium (0–40%) range PHB content. However, additional reference methods (*e*.*g*., carbohydrates or carboxylic acids) should be included in the multivariate analysis and prediction model to fully characterize differences in the chemical composition and improve the model’s predictive power.

### Carbon substrate phenotypic assay by biolog PM1

To aid in future studies such as strain characterization by high-throughput screening methods, we additionally tested carbon substrate utilization by *Halomonas* sp. MC140 in Biolog PM1 plates.

Biolog is a widely used assay for determining a strain’s carbon source phenotype and screening for bio-industrial potential^11,53^. The PM1 assay plate contains 95 different carbon substrates distributed in separate wells, each containing a dye added that changes color upon chemical reduction by bacterial respiration^54,55^. Spectrophotometric measurement of this color change, relative to a control well without an added carbon source, allows for the determination of the bacterial phenotype.

**Fig. 4:**
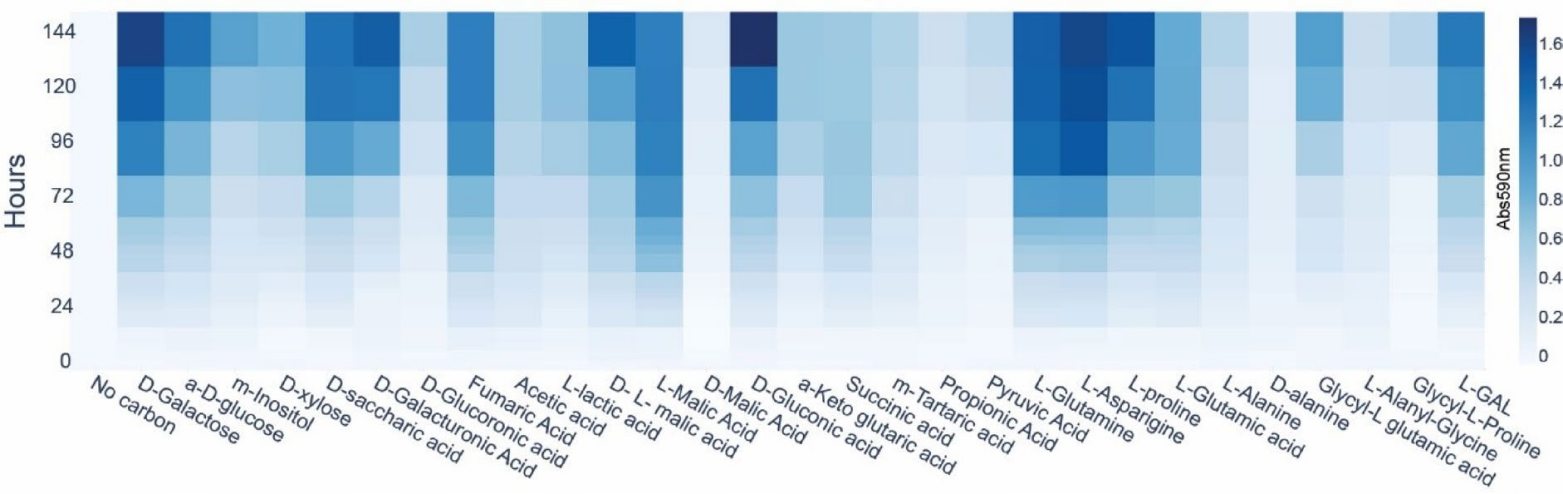
Biolog substrate utilization phenotype for *Halomonas* sp. MC140. The heat map shows the Biolog PM1 substrate utilization measured as absorbance at 590 nm and shown by increased color intensity for 29 selected substrates and the no carbon control (*n* = 3). The substrate L-GAL corresponds to L-Galactonic Acid-g-Lactone.

Based on this assay, 29 substrates were found to be metabolized by *Halomonas* sp. MC140, as defined by exceeding an arbitrary threshold of 0.3 absorbance units above the “No carbon” well. Several of these 29 substrates elicited strong respirational response by *Halomonas* sp. MC140 in the Biolog PM1 plates including sugars and organic acids as shown in a heatmap in Fig. 4. The carbon substrate utilization phenotype was furthermore compared to that of the Arctic isolate *Halomonas* sp. R5-57^30^. Three substrates, D-saccharic acid, a-Keto glutaric acid, and m-Tartaric acid were metabolized only by *Halomonas* sp. MC140, 26 substrates were metabolized by both and 22 metabolized only by *Halomonas* sp. R5-57 (Supplementary Table S3). Among the 22 substrates metabolized only by *Halomonas* sp. R5-57 was glycerol. As described previously, *Halomonas* sp. R5-57 were better at utilizing glycerol for production of PHA compared to *Halomonas* sp. MC140.

Several carbon sources utilized by the Arctic isolates *Halomonas* sp. MC140 and *Halomonas* sp. R5-57 are promising for bio-industrial production of PHA and other biomolecules. These include acetic acid (acetate), hexoses like galactose and glucose, and pentoses like xylose (Fig. 4 and Supplementary Table S3). The ability to metabolize these specific carbon sources is interesting, because they are all found in spent sulphite liquor (SSL), a byproduct of the wood industry. The potential of using SSL for production of PHA using *Halomonas* species with different carbon source phenotypes was investigated by others^56^. The substrate utilization phenotype described for *Halomonas* sp. M140 by use of the Biolog PM1 assay may therefore serve as a starting point for future research, such as testing mixed industrial waste substrates with known carbon sources, e.g. in small-scale cultivation setups paired with high-throughput FTIR screening for PHA production.

### Implications and future directions

The *Halomonas* sp. MC140 strain was found to produce up to 35 ± 4.8% PHB when fed with acetate in media that contained approximately similar amounts of salts as seawater. Although the growth temperature profile was mesophilic, the strain is clearly psychrotolerant as it was isolated from a cold Arctic littoral environment in Norway. The relatively low production of PHA compared to other *Halomonas* makes it less likely to use this strain for industrial bioplastic production. However, another potential application could be for use as feed in aquaculture. Norway has a large aquaculture production of Atlantic Salmon (*Salmo salar*), which exceeded 1.4 million tons from supplementing approximately 2 million tons of feed, in 2020^57^. This feed comes from marine sources and land-based agriculture and is the main negative environmental impact factor with regards to CO_2_ emissions^58^. To further reduce the environmental and economic cost of aquaculture, use of alternative waste materials is envisioned. For this purpose, several studies have demonstrated that bacteria containing PHA can be used as feed in various types of aquaculture^59^. Identification and testing Norwegian waste resources for bacterial PHA production in strains and mixed microbial cultures capable of growth in conditions similar to *Halomonas* sp. MC140, for example with regards to salt tolerance, could pave the way for improving sustainability in the Norwegian Atlantic Salmon farming industry.

## Conclusion

The strain *Halomonas* sp. MC140 was isolated from an Arctic environment and WGS revealed the presence of PHA biosynthesis genes like those observed in other members of the *Halomonas* genus. Phylogenetic analyses based on 16 S rRNA and WGS proteome data, generated using the TYGS pipeline, identified the strain as most closely related to *Halomonas profundi* MT13 (*Vreelandella* cf. *profundi*). Polyhydroxyalkanoates were produced in the form of PHB and PHBV, with PHB production reaching up to 35 ± 4.8% PHB when acetate was supplied as carbon source. The carbon substrate utilization phenotype of *Halomonas* sp. MC140 was characterized using the Biolog PM1 assay and serves as a starting point for future optimization of PHA production in this strain. Additionally, multivariate FTIR analysis were used to construct a PLSR model for predicting PHB content in *Halomonas sp. MC140* in the low to medium (0–40%) range. These findings highlight the potential of using predictive modeling by FTIR for further optimization of PHA production in *Halomonas sp. MC140* and its possible applications in sustainable biotechnology, including aquaculture feed or bioplastic production.

## Methods

### Strain isolation and PHA pre-screening on agar plates

*Halomonas* sp. MC140 was isolated from a maturation room for salted fish at a fish-landing facility outside Tromsø, Norway. Environmental swabs were plated on M4 agar plates ^27^, and distinct colonies were incubated in 3 mL M4 media in 12 mL tubes at 15 °C, with shaking at 200 rpm. This procedure was repeated several times to obtain pure colonies. The pure isolate was stored in 20% glycerol (VWR) at −80 °C and revived on MA (Difco 2216) plates at 25 °C. The strain was pre-screened for PHA production on MMY plates^27^ supplemented with 20 g/L glucose (VWR) and 15 g/L agar powder (VWR) and were incubated for 72 h at 14 °C before sampling by FTIR.

### DNA extraction and WGS

Extraction of DNA, WGS, and genome assembly followed the workflow previously described^27^, but with the following settings and updated software versions run in Python 3.11.2: Trimmomatic 0.39 for read trimming (Phred33, minlen 30, leading 5, trailing 10, Illuminaclip, Nextera3: 2:30:10)^60^, BBduk (ftm 5, Ktrim R, K 23, Mink 7, Hdist 1, Tbo and Tpe), BBmerge and BBnorm (Prefilter true, target 40, min 5 and error correction settings ecc t, keepall passes 1, bits 16) from BBmap 39.01 for quality control and normalization^61^, Kraken2 2.1.1 for contamination control by taxonomic classification visualized by KronaTools 2.8.1^62–64^, and SPAdes 3.15.5 for assembly^65^. Genome completeness was assessed with BUSCO 5.4.6 using the oceanospirillales_odb10 database^66–68^. Assembly quality was evaluated with Quast 5.0.2^69,70^. Read mapping was performed with Bowtie2 2.4.5 and Samtools 1.11^71,72^.

The assembled genome was annotated with the NCBI Prokaryotic Genome Annotation Pipeline^73^ and compared with *Halomonas* sp. R5-57 (NCBI acc. LN813019) using with The Kyoto Encyclopedia of Genes and Genomes (KEGG) BlastKOALA 3.0 (https://www.kegg.jp/blastkoala)^74^. The KEGG output files were compared using the Set function in Pandas 2.0.1 within Python 3.8.5.

### Phylogenetic classification

The 16S rRNA region was initially sequenced to identify the isolate to genus level using genomic DNA amplified by PCR^27^. The assembled genome was later submitted to DSMZ’s Type (Strain) Genome Server (TYGS) (https://tygs.dsmz.de/, 2023-04-13), which constructs 16S rRNA and proteome-based phylogenies, alongside calculations of guanine-cytosine content, DNA-DNA hybridization (DDH), and genome-based species delimitation via Genome Blast Distance Phylogeny^75–81^. The 10 most closely related type strains were determined using the MASH algorithm for intergenomic relatedness and sequence extraction via RNAmmer and BLAST^82–84^. Genome BLAST Distance Phylogeny (GBDP) employed the ‘coverage’ algorithm and distance formula d5 for phylogenomic inferences and pairwise comparisons, with 100 distance replicates^80^. Proteome analysis was also performed using GBDP. Digital DDH (dDDH) values and confidence intervals were calculated using the Genome-to-Genome Distance Calculator 3.0^76,80^. The intergenomic distances were used to infer a balanced minimum evolution tree with branch support using FASTME 2.1.6.1, including SPR postprocessing^85^, and 100 bootstrap replicates for branch support. The trees were midpoint rooted^86^, with species and subspecies clustering based on 70% and 79% dDDH thresholds, respectively^75,87^.

### Sequence alignment and amino acid similarity

The PhaB sequence from *Halomonas* sp. MC140 was aligned with *H. bluephagenesis* TD01 PhaB obtained from NCBI (WP_009724067.1) using CLC MainWorkbench 21.0.2. Amino acid similarities were determined by querying sequences from *Halomonas* sp. MC140 against the non-redundant protein database using Protein Basic Local Alignment Search Tool (BLAST)^88^ (blastp) from the National Center for Biotechnology Information’s server (https://blast.ncbi.nlm.nih.gov/Blast.cgi).

### PHA production

Starter cultures for PHA production were prepared by transferring colonies from MA plates into Lysogeny broth^89^ (LB_3 –5_) media composed of 5 g/L yeast extract (Merck), 10 g/L tryptone (Sigma-Aldrich), and 35 g/L sodium chloride (VWR). Three individual colonies were cultivated at 25 °C, 200 rpm, in three successive steps from 3 ml to 50 ml to 250 mL, each step reaching middle of the exponential growth phase. At the final step, when reaching an optical density at 600 nm (OD_600nm_) of 3–4, 45 ml were centrifuged 10 min at 11,000 *g*, re-suspended into PHA production medium, and then grown at same conditions as the starter cultures for 120 h. Another batch of glucose and glucose supplemented with propionate was extended to 168 h. The PHA production medium contained (per liter) 3.00 g KH_2_PO_4_, 0.20 g NH_4_Cl, 6.00 g Na_2_HPO_4_ · 2 H_2_O, and 31.5 g NaCl. Carbon substrates were supplied in a carbon to nitrogen ratio of 50:1, with carbon calculated as acetyl-CoA equivalents relative to the nitrogen content (3.7 mM)^30^. The substrates and macronutrients were added separately as follows (final concentration indicated): sodium acetate (21.3 g/L acetate), glycerol (13.7 g/L), magnesium sulfate (2 mM), and calcium chloride (1 mM) were autoclaved, while glucose (16.8 g/L), fructose (16.8 g/L), and sodium hydroxide neutralized propionic acid (propionate, 0.03 M) were sterile filtered (0.2 μm).

### Quantification of PHA by GC-FID analysis

The amount of PHA was quantified by GC-FID as previously described^30^ and expressed as percentage of the CDW by gravimetry of lyophilized cell pellets measured on an analytical scale.

### FTIR spectroscopy

Colonies from agar plates were scraped off with an inoculation loop, washed in 200 µL methanol (VWR), and separated by centrifugation at 18,000 g for 3 min in Eppendorf tubes. Similarly, biomass from 500 µL of liquid culture from different time-points were harvested by centrifugation under the same conditions but washed with 35 g/L sodium chloride (VWR) and centrifuged again. All samples were analyzed on an Agilent Cary 630 FTIR spectrometer, with instrument settings as previously reported^27,30^.

### Multivariate spectral analysis and predictive modeling

The FTIR spectra sampled from the final step of LB preculture and from supplement with pure substrates at the endpoint of shake incubation (*n* = 34) were pre-processed and analyzed by PCA using Orange Spectroscopy v. 3.36.2 within Python v. 3.8.18. The spectra underwent second derivative transformation using the Savitzky-Golay algorithm^90,91^ (window size 11 and second polynomial order), removal of uninformative regions (< 900, 1810–2860, and > 3050 cm^− 1^), and extended multiplicative signal correction (EMSC) with linear and quadratic terms^92^. A reference spectrum for EMSC was established from the mean of six spectra obtained from the starter cultures of *Halomonas* sp. MC140 grown in LB_3 − 5_ media. A reference spectrum for PHBV was obtained from a 12 mol % PHBV pellet (Sigma-Aldrich). The dataset used for predictive modeling was furthermore subjected to amide band I normalization (i.e. peak normalization at 1652 cm^− 1^)^51^. Predictive modeling by PLSR was done with Aspen Unscrambler v. 14.2. The model was constructed with the endpoint samples from 120 h (*n* = 22) with the pre-processed and amide band-I region normalized FTIR spectra used as predictor (X-variables) and GC-FID as responses (Y-variables) and internally validated by full cross validation. Optimal number of PLSR components (i.e. PLSR factors) of the calibration models (*A*_*Opt*_), root-mean-square error (RMSE) and coefficient of determination (R^2^) were calculated, and the optimal model was selected based on the lowest *A*_*Opt*_ having insignificantly higher RMSE than the model with the minimum RMSE. For the independent test set validation, the glucose supplemented samples (with and without propionate) harvested after 168 h (*n* = 11) were used.

### Biolog screening

The Biolog assay was conducted as three independent experiments each consisting of one Biolog PM1 plate (Biolog, USA) in Biolog PM1 minimal medium (MM_PM1_) as described previously^30,93^.

### Data analysis

The phylogenetic trees were visualized with ITol 6.7.4^94^. Figures containing FTIR and Biolog data were created using Python 3.10.13 with the following libraries: Pandas 2.1.4 for data preparation, Plotly 5.13.0 for visualization, and Statsmodels 0.14.0 for linear regression.

## Electronic supplementary material

Below is the link to the electronic supplementary material.

Supplementary Material 1

## Data Availability

The Whole Genome Shotgun project has been deposited at GenBank under Biosample acc. SAMN17527550, with sequencing reads deposited under NCBI BioProject accession PRJNA678481 and in the NCBI Sequence Read Archive under acc. SRR13530298.The FTIR and GC-FID dataset used for multivariate analysis and predictive modeling is openly available in Zenodo under the dataset titled “Halomonas sp. MC140 - FTIR spectra of bacterial cells and GC-FID quantification of PHA” accessible at https://doi.org/10.5281/zenodo.15175258.

## Abbreviations

3-HV: 3-hydroxyvalerate
C: Cytosine
CDW: Cell dry weight
DDH:DNA: DNA hybridization
dDDH: Digital DDH
LB: Lysogeny broth
EMSC: Extended multiplicative signal correction
FTIR: Fourier transform infrared spectroscopy
G: Guanine
GC-FID: Gas chromatography-flame ionization detector
HA: Hydroxyalkanoic acid
GBDP: Genome BLAST distance phylogeny
KEGG: The Kyoto Encyclopedia of Genes and Genomes
Lcl: Long chain length
Mcl: Medium-chain length
NADH: Nicotinamide adenine dinucleotide
NADPH: Nicotinamide adenine dinucleotide phosphate
OD_600nm_: Optical density at 600 nm
PHA: Polyhydroxyalkanoates
PHB: Polyhydroxybutyrate
PHBV: Polyhydroxybutyrate-co-valerate
PC: Principal component
PCA: Principal component analysis
PLSR: Partial least square regression
R^2^: Coefficient of determination
RMSE: Root mean square error of prediction
Scl: Short-chain length
SSL: Spent sulphite liquor
TYGS: Type (strain) genome server
WGS: Whole genome sequencing
