# Supplementary material for: Halomonas sp. MC140, a polyhydroxyalkanoate (PHA) producer isolated from the Arctic environment
Source: Sci Rep. 2025 Jul 3;15:23744. doi: 10.1038/s41598-025-06898-7 (PMC12229310; doi:10.1038/s41598-025-06898-7)
Supplement: Supplementary file 1 — Supplementary Material 1 [file 41598_2025_6898_MOESM1_ESM.docx]

| **Supplementary Table 1.** Genes encoding enzymes involved in propanoate metabolism, methyl-citrate cycle and the glyoxylate shunt. The amino acid (AA) identity is relative to *H. profundi* MT13, except a: *Halomonas neptunia*,  b: unclassified *Halomonas*. | | | | | |
| --- | --- | --- | --- | --- | --- |
| **Pathway** | **Gene(s)** | **Protein function (EC.)** | **KEGG id** | **Locus Tag** | **AA identity (%)** |
| Propanoate metabolism | *ackA* | Acetate/propionate family kinase  (EC. 2.7.2.1) | K00925 | JNO04_10885 | 97.0^a^ |
|  | *ACSS1_2* | 1: AMP-binding protein  2: Acetate--CoA ligase (EC. 6.2.1.1) | 1 and 2: K01895 | 1: JNO04_09805 2: JNO04_11415 | a) 98.6 b) 98.6^b^ |
|  | *pta* | bifunctional enoyl-CoA hydratase/phosphate acetyltransferase (EC. 2.7.2.1) | K00625 | JNO04_10880 | 98.3^a^ |
|  | *ACSS3, prpE* | Propionyl-CoA synthetase (EC. 6.2.1.17) | K01908 | JNO04_08360 | 97.7 |
| Methyl-citrate-cycle | *prpC* | 2-methylcitrate synthase (EC. 2.3.3.5) | K01659 | JNO04_10035 | 98.7^b^ |
|  | *prpD* | Bifunctional 2-methylcitrate dehydratase/aconitate hydratase (EC. 4.2.1.79) | K01720 | JNO04_10020 | 97.8 |
|  | *acnB* | Bifunctional aconitate hydratase 2/2-methylisocitrate dehydratase (EC. 4.2.1.3) | K01682 | JNO04_14905 | 99.9 |
|  | *prpB* | Methyl isocitrate lyase (EC. 4.1.3.30) | K03417 | JNO04_10040 | 98.7^b^ |
|  | *sucD* | Succinyl-CoA synthetase alpha subunit  (EC. 6.2.1.5) | K01902 | JNO04_05495 | 100 |
| Glyoxylate shunt | *gltA* | Citrate synthase (EC. 2.3.3.1) | K01647 | JNO04_05450 | 100 |
|  | *acnA* | aconitate hydratase (EC. 4.2.1.3) | K01681 | JNO04_04305 | 99.7 |
|  | *Iclb* | Isocitrate lyase (EC. 4.1.3.1) | K01637 | JNO04_05615 | 98.3 |
|  | *aceB* | Malate synthase (EC. 2.3.3.9) | K01638 | JNO04_07600 | 98.8 |

| **Supplementary Table 2.** List of carbon substrates metabolized by the Arctic isolates *Halomonas* sp. MC140 and *Halomonas* sp. R5-57 as determined by the Biolog assay. | | | |
| --- | --- | --- | --- |
| **Metabolized by** | ***Halomonas* sp. MC140** | ***Halomonas* sp. R5-57** | **Both** |
|  | D-saccharic acid | 2-Aminoethanol | Acetic acid |
|  | a-Keto glutaric acid | Bromo Succinic Acid | D- L- malic acid |
|  | m-Tartaric acid | Citric Acid | D-Malic Acid |
|  |  | D-Cellobiose | D-Galactose |
|  |  | D-Fructose | D-Galacturonic Acid |
|  |  | D-Galactonic acid - D-lactone | D-Gluconic acid |
|  |  | D-Mannitol | D-Glucoronic acid |
|  |  | D-sorbitol | D-alanine |
|  |  | D-trehalose | D-xylose |
|  |  | Glycerol | Fumaric Acid |
|  |  | L-arabinose | Glycyl-L glutamic acid |
|  |  | L-aspartic acid | Glycyl-L-Proline |
|  |  | Lactulose | L-Alanine |
|  |  | Maltose | L-Alanyl-Glycine |
|  |  | Maltotriose | L-Asparigine |
|  |  | Methyl Pyruvate | L-Galactonic Acid-g-Lactone |
|  |  | Mono Methyl Succinate | L-Glutamic acid |
|  |  | N-acetyl-D-glucosamine | L-Glutamine |
|  |  | Sucrose | L-Malic Acid |
|  |  | Thymidine | L-lactic acid |
|  |  | a-D-Lactose | L-proline |
|  |  | b-Methyl-D-Glucoside | Propionic Acid |
|  |  |  | Pyruvic Acid |
|  |  |  | Succinic acid |
|  |  |  | a-D-glucose |
|  |  |  | m-Inositol |


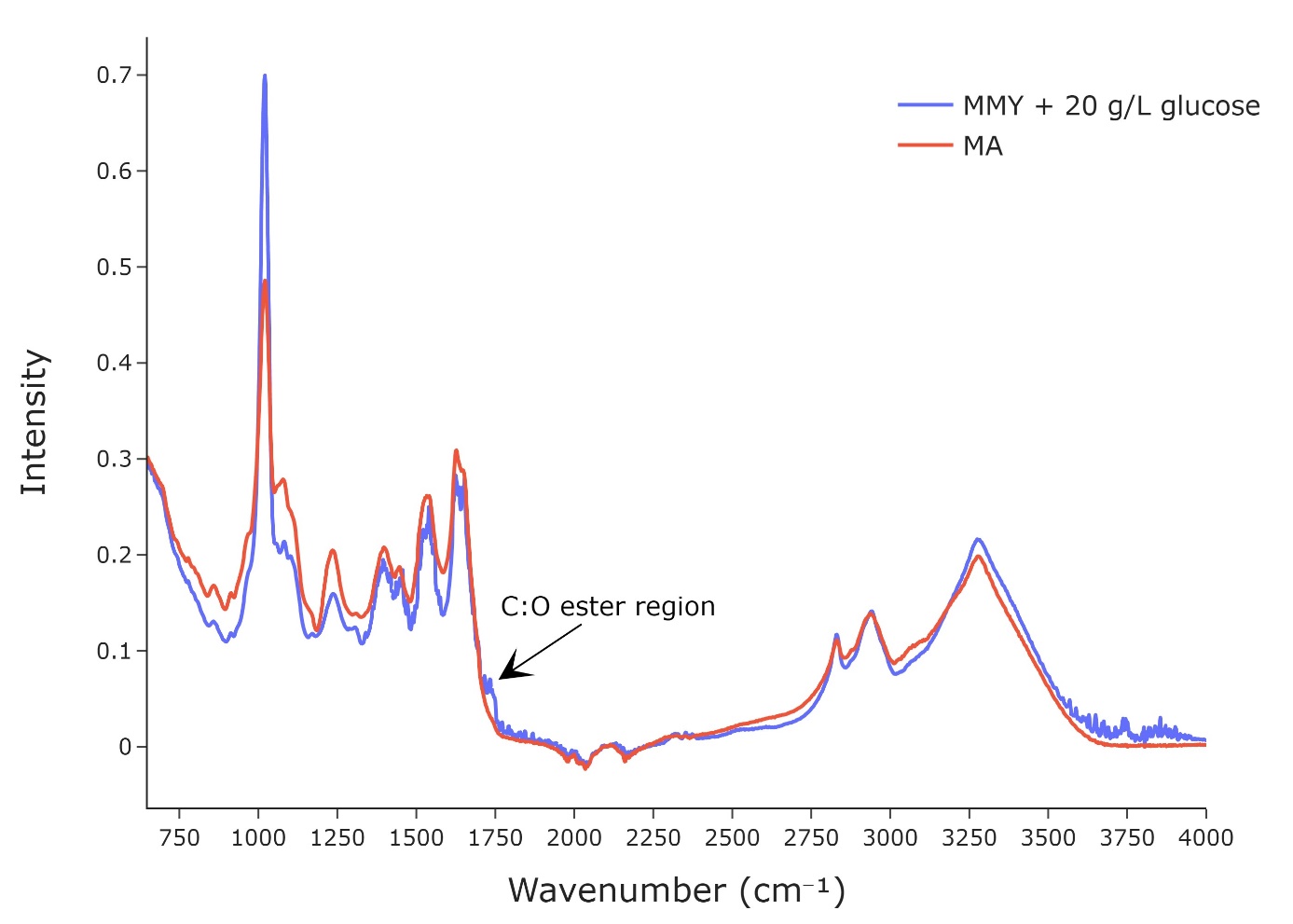


Supplementary Figure 1. Amide-peak (1652 cm^-1^) normalized FTIR spectra of *Halomonas* sp. MC140 colonies sampled after four days growth at 14 °C from MMY agar plates supplemented with 20 g/L glucose or MA plates. The carbonyl-ester regions peaks are indicated by the arrow.


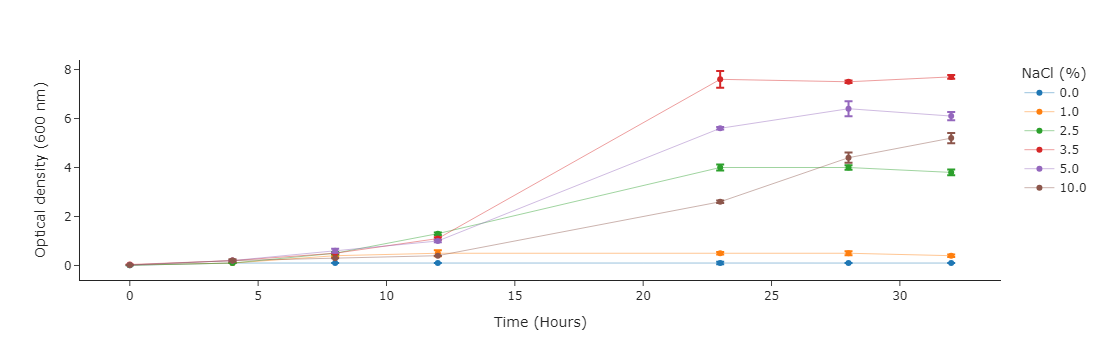


Supplementary Figure 2. Growth of *Halomonas* sp. MC140 measured by optical density in LB media at 25 °C, 200 rpm, with different sodium chloride concentrations.


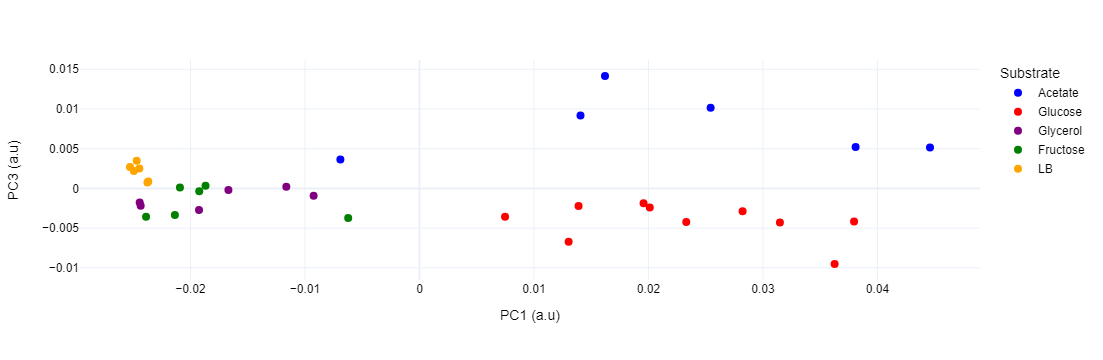


Supplementary Figure 3. The score values of the third principal component (PC3) plotted against the first principal component (PC1). Separation between acetate and glucose supplemented samples is observed in PC3.


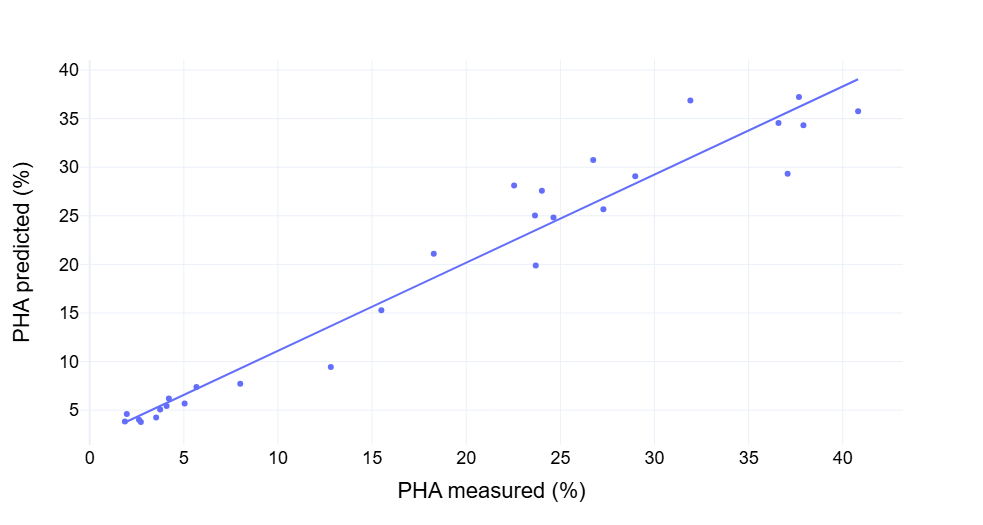


Supplementary Figure 4. The predicted values for PHA content in *Halomonas* sp. MC140 samples based on the PLSR model of FTIR spectra and GC-FID reference values. The independent test set coefficient of determination (R^2^) were 0.97 and root mean square error (RMSE) of prediction were 2.26 %.
